# Wording Error in Introduction

**DOI:** 10.1001/jamahealthforum.2022.3770

**Published:** 2022-10-07

In the Viewpoint titled “Federal and State Pharmacy Regulations and Dispensing Barriers to Buprenorphine Access at Retail Pharmacies in the US,”^1^ published August 26, 2022, there was a wording error in the introduction. The phrase given as “Nearly 20% of individuals with OUD are not treated with a MOUD” should have omitted the word “not” to read “Nearly 20% of individuals with OUD are treated with a MOUD.” This Viewpoint has been corrected.
